# Advanced real-time detection of acute ischemic stroke using YOLOv12, YOLOv11, and YOLO-NAS: a comparative study for multi-class classification

**DOI:** 10.1038/s41598-025-18997-6

**Published:** 2025-09-15

**Authors:** Marwa El-Geneedy, Hossam El-Din Moustafa, Hatem Khater, Seham Abd-Elsamee, Samah A. Gamel

**Affiliations:** 1 Electronics and Communications Engineering Department, Mansoura University, Mansoura, 35516 Egypt; 2 Computer and Systems Engineering Department, Faculty of Engineering, Horus University-Egypt, New Damietta, 34518 Egypt; 3 Faculty of Engineering, Horus University-Egypt, New Damietta, 34518 Egypt; 4 Dean of Faculty of Artificial Intelligence and information, Horus University-Egypt, New Damietta 34518, Egypt

**Keywords:** Stroke, Real-time detection, YOLOv12, YOLOv11, YOLO-NAS, Multi-class classification, Medical imaging, Computational biology and bioinformatics, Engineering, Health care, Mathematics and computing, Medical research

## Abstract

Acute ischemic stroke (AIS) remains a leading cause of mortality and disability worldwide, demanding diagnostic tools that are both accurate and fast for timely intervention. This study presents a comparative evaluation of three state-of-the-art object detection models-YOLOv12, YOLOv11, and YOLO-NAS-for multi-class AIS detection in magnetic resonance imaging (MRI). The dataset, comprising four categories (Normal, PD-Patient, Acute Ischemic Stroke, and Control), was preprocessed with normalization, resizing, and augmentation, then split into training (70%), validation (20%), and testing (10%). Models were trained and evaluated on identical data, with performance measured by precision, recall, mean average precision at IoU 0.5 (mAP@50), and inference speed. YOLOv11 achieved the highest mAP@50 (98.5%) and balanced precision (95.4%) and recall (96.6%), making it the most reliable across classes. YOLOv12 performed comparably (mAP@50 98.3%, precision 95.2%, recall 96.0%) with slightly slower inference, while YOLO-NAS offered the fastest speed (154 FPS) but lower precision (76.3%. Results highlight the trade-offs between detection accuracy and processing speed, providing guidance for selecting YOLO-based architectures suited to specific clinical workflows such as emergency stroke care. The real-time implementation, accessible via Roboflow, demonstrates the feasibility of deploying these models for rapid, automated AIS detection in clinical settings.

## Introduction

Stroke is a common cause of morbidity and mortality worldwide. One in four individuals over the age of 25 may have a stroke over their lifetime, with over 12.2 million new stroke cases reported year globally. The ischemic subtype of stroke (AIS) constitutes almost 62% of all global occurrences^1^. Medical imaging has historically propelled the development of advanced categorization, detection, and segmentation systems. Instance segmentation has been thoroughly examined in the context of brain strokes, especially in magnetic resonance imaging (MRI), which is prevalent the scientific literature. On the other hand, segmentation in MRI has garnered far less effort, with research on its use in real-time intraoperative environments being very rare^2^. Machine learning, a significant subset of artificial intelligence, has shown substantial promise in recent advancements in medicine, using machine learning methodologies to significantly improve the efficacy of prediction models^3^. Brain imaging is essential for the diagnosis and prognosis of AIS. The emergence of deep learning algorithms has transformed medical image analysis to the extent that abnormalities can be automatically detected without pixel-level accuracy.

Object detection has been recognized as a key solution for real-time analysis in fast-paced sectors such as agriculture, transportation, education, and healthcare. One of the most notable algorithms is “You Only Look Once” (YOLO) by Redmon et al.^4^, which has transformed real-time detection by merging area proposals and classification into one neural network. The latest versions of YOLO, including YOLO NAS, YOLO11, and YOLOv12, epitomize the highest level of the model’s improvement^5^. Newer advancements on the YOLO family models include YOLOv11, YOLOv12, and YOLO-NAS; each of these contributed uniquely to real-time object detection. YOLOv11 demonstrates architectural innovations with increased accuracy and efficiency at a trade-off in their detection performance versus their computation. YOLOv12, while architecturally somewhat deeper than its predecessors, introduced greater computational overhead and did not produce an appreciable performance improvement^6^. YOLO-NAS incorporated the NAS generating method to realize the functionality of automating model design with respect to the trade-off between accuracy and resource consumption enhancement. Their advancements show the way YOLO architectures have progressed, and researchers are always seeking better real-time performance in various applications^7^.

The purpose of the research is to conduct an extensive analysis that compares the evolution of the YOLO algorithm. This research makes significant contributions to the field through its comprehensive evaluation of YOLOv12 which represents the newest member of the YOLO family. The study evaluates multiple performance criteria that include accuracy, precision and recall, and mean average precision (mAP). Furthermore, we examine the practical uses of each YOLO variant, highlighting their advantages and drawbacks in various use scenarios. The research comparison aims to deliver essential findings that improve the efficient use of these models in various contexts for researchers and practitioners.

Stroke rates are still among the top causes of death and disability in the world, as well as acute ischemic stroke (AIS) which constitutes around 62% of all stroke patients. Early diagnosis is essential, as it enhances the outcomes of the patient to a high degree. The role of Magnetic Resonance Imaging (MRI) follows, especially modalities, referred to as diffusion-weighted imaging (DWI) and fluid-attenuated inversion recovery (FLAIR). Nevertheless, the prompt interpretation of these images has continued to present a challenge in most patient care situations and particularly in case of time limit or in places with limited resources. Deep learning has been considered an influential means to analyze medical images such as detection, segmentation, and classification of the image. Though many experiments investigated CNN-based frameworks and preimplantation of YOLO (e.g., YOLOv3 to YOLOv8) regarding stroke-related detection, there is a gap in terms of benchmarking the most advanced creativity of YOLO in terms of AIS.

This paper resolves this gap by providing a comparative analysis in real-time of YOLOv12, YOLOv11, and YOLO-NAS deployed to detect AIS in multi-class MRI images in great detail. Both of the models are major architectural improvements on the YOLO family:

- YOLOv12 combines the mechanisms of attention such as Area Attention and Flash Attention/ or is able to provide experience close to real-time inference speeds.
- YOLOv11 is an improvement of YOLOv8 using Cross Stage Partial Self-Attention (C2PSA) as means of feature propagation.
- YOLO-NAS uses Neural Architecture Search (NAS), and quantization-aware modules to achieve a trade-off between detection performance and device computational efficiency at the edge.
- Comparative performance of YOLOv12, YOLOv11, and YOLO-NAS on AIS detection on MRI data was analyzed as a first-time attempt.
- Multi-class detection, where Normal, PD-patient, Acute Ischemic Stroke, and Control classes are considered ?not a classical binary classification.
- A stringent metric containing precision, recall, mAP@0.5, confusion matrices, and dynamics to deploy in the real-life environment via an online accessible interface (Roboflow).
- Evaluation using an external dataset in addition to internal validation data to evaluate the generalizability of performance under variant imaging conditions and with different patient populations.

The following article is structured as follows: Section 2 Literature Review. Section 3 presented the Material and Methods, and Section 4 presents the Results, followed by Section 5 which provides the Discussion and comparative analysis with related studies. Section 6 addresses the study’s Limitations and outlines Future Work directions. Finally, Section 7 presents the Scope and Conclusions.

## Literature review

YOLO (You Only Look Once) stands as an elite approach for object detection processes. The system delivers top-notch speed while delivering accurate results so it finds wide application in numerous sectors. Research about this object identification method increased after academics began publishing articles that studied its development and model refinement and performance comparison against alternative computer vision algorithms. Variety of analyses about YOLO demonstrates its tremendous influence on the progress of computer vision.

Nasser et al.^8^ developing a deep-learning-based framework for recognizing acute ischemic stroke (AIS) using MRI and CT brain images. The image enhancement, feature extraction, and fine-tuned classification with residuals and split attention-based network (ResNest) are the three phases that made the framework. The quality of images is improved using preprocessing involving a generative adversarial networks (GANs), and features are extracted using YOLOv7. The fine-tuning of the hyperparameters of the ResNest model is done using Aquila optimization. Two benchmark datasets are used: MRI images (1021 healthy, 955 unhealthy) and CT images (1551 healthy, 950 unhealthy). It provides an accuracy and F1 score of 98.25 and 97.28 for MRI images and 98.65 and 98.25 for CT images, respectively, outshining earlier methods. Plus, it also ensures lightweight performance; thus, its application in the real-world healthcare system looks promising. This work shows great promise for the use of deep learning in improving the diagnosis of AIS, thereby lessening the burden on healthcare systems.

Another research introduced by Ya-Fang et al.^9^ focuses on developing a deep learning framework for automatic segmentation of carotid plaques from MRI scans to assess stroke risk. The pre-trained model architectures used in this study include YOLOv3, MobileNet, and RCNN; these models were fine-tuned for this specific task. They attained high classification accuracies via the study methodology: 94.81%, 92.53%, and 90.23% for YOLOv3, RCNN, and MobileNet, respectively. The dataset included MRI scans from 265 patients involving extensive description of the inclusion criteria and scanning parameters.

Shannan et al.^10^ introduces a deep learning framework, TE-YOLOv5, to enhance the detection of stroke lesions in diffusion-weighted imaging (DWI). These radiologist-challenging, often small stroke lesions are typically quite difficult to locate and delineate due to their ill-defined boundary. This TE-YOLOv5 model integrates Technical Aggregate Pool(AP) and Reverse Attention(RA) modules to boost the performance in feature extraction and edge tracing. A total of 1681 DWI images were enrolled in this study of 319 stroke patients; of those, 80% were allocated to a Training dataset, while the remaining 20% were reserved for Testing. TE-YOLOv5 outperformed baseline YOLOv5 models with 81.5% precision, 75.8% recall, and 80.7% mAP@0.5. Ablation studies indicated the significant contributions of both AP and RA modules since they severely degraded performance metrics upon being disabled. The model also reached a 98.51% positive finding rate at the patient level when the confidence threshold was 80%. Needful help that TE-YOLOv5 could provide to radiologists by making allowance for lower rates of misdiagnosis and boosts detection of strokes in real-time could be underscored from this work.

Abdussalam et al.^11^ introduced a way to detect stroke based on deep learning and federated learning methods. In the presented study, stroke symptoms based on facial paralysis will be detected in real time using YOLOv8, which is a state-of-the-art neural network architecture. Federated learning is introduced as a mechanism by which learning can be done while ensuring privacy preservation: You can decentralize the training process across various clients, enabling the model to learn in a way that does not compromise sensitive data about patients. The methodology takes NVIDIA platforms into account for implementation so that efficient processing can be performed on any edge device with GPU capabilities. The methodology employs YOLOv8 models, a cutting-edge neural network architecture, to identify stroke cases based on facial paralysis from images.

Suleyman et al.^12^ suggested a deep learning-based system using models such as YOLOv7, YOLOv8, and YOLOv9 to automatically detect, localize, and segment ischemic and hemorrhagic strokes in brain CT images. It uses a dataset of 6,951 slices from the Turkish Ministry of Health, which were preprocessed and augmented. Among the group, YOLOv9-Seg obtained the highest performance in ischemic stroke 99.50%, hemorrhagic stroke 99.49%, and both combined 99.71%. On the whole, YOLO models were superior in accuracy and real-time processing than traditional U-Net-based models. A user-friendly app was built in Streamlit to help medical professionals make a diagnosis of strokes fast. The study presents YOLOv9-Seg to be the best model, which gives a chance to AIl-assisted stroke diagnosis in clinic settings.

Maram et. al.^13^ like other studies, the authors use YOLOv5 and YOLOv7 to detect and delineate tumor from MRI images. Three tumor types are studied: meningioma, glioma, and pituitary tumors-from 3064 MRI images. The authors used mask alignment techniques in their segmentation, where the models will be compared based on precision, recall, mAP, and F1-score. The results revealed that YOLOv5 reached a mAP@0.5 of 94.7%, whereas YOLOv7 outperformed it with a mAP@0.5:0.95 of 67.7%. YOLO-based models proved more efficient than traditional segmentation paradigms such as RCNN, Faster RCNN, and Mask RCNN. Yet, a significant class of false positives arose within glioma segmentation despite high accuracy rates. The work elucidates the possibility of real-time, highly accurate MRI brain tumor segmentation and classification owing to the unique characteristics of YOLO-based architectures.

This paper will presents many significant contributions:

- The proposed research incorporates advanced YOLO v2 and v1 and NAS versions to design a rapid and reliable multi-class acute ischemic stroke detection system. The framework functions quickly and accurately to analyze CT and MRI scans suitable for deployment in clinical establishments which need fast diagnostic services.
- This proposed framework possesses the capability to modify the diagnosis process of strokes through shorter diagnostic durations and superior care results. The system demonstrates versatility in clinical applications by effectively diagnosing four stroke types: normal responses as well as Parkinson’s disease (PD) cases and acute ischemic stroke and control groups.
- The study conducts extensive performance evaluation using confusion matrices and precision-recall curves and mAP metrics at different Intersection-over-Union (IoU) threshold points. The chosen metrics offer a comprehensive understanding of how well the model performs thus providing both transparency and the ability to reproduce results. Training and validation loss graphs provide visual insights into YOLO versions’ learning behaviors which help improve their performance.
- A real-time application accessible through Roboflow provides an end-to-end platform which allows healthcare professionals along with researchers to process and obtain predictions for brain scans with simplified interface access. The easy-to-use interface of this platform gives medical personnel and researchers basic access to the latest AI technologies so they can apply them within research operations and clinical procedures.
- The designed model underwent complete evaluation through testing mechanisms on internal test data and external datasets acquired from Kaggle. The model demonstrates its reliable performance capabilities by maintaining consistency between analyzed known and unknown imaging data which solves one main obstacle medical AI systems face when dealing with various scanning conditions and different patient groups.

The research presented in this paper establishes connections between modern deep learning technology with realistic healthcare diagnostic practices. The acute ischemic stroke detection system establishes present and future standards in AI-driven diagnostics through its precise real-time approach and provides accessible healthcare technology foundations.

## Material and methods

### Data-set and pre-processing

**Fig. 1:**
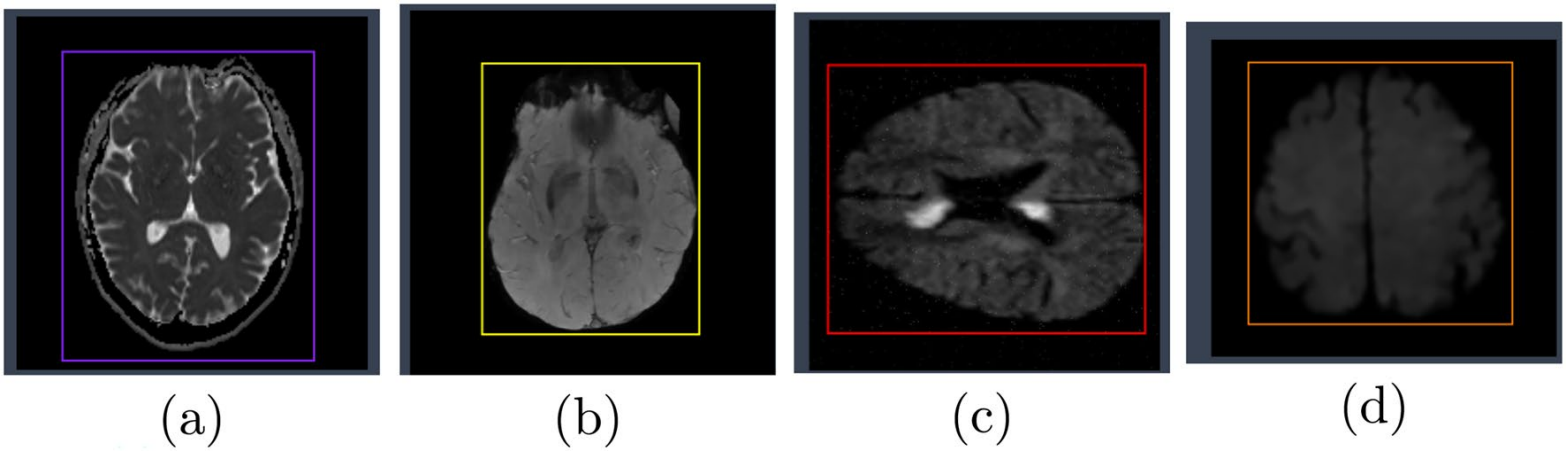
Samples of dataset for (**a**) normal, (**b**) PD-patient, (**c**) acute-ischemic-stroke, and (**d**) control.

The dataset has been organized by Hrushikesh and is hosted on the Roboflow platform. It is an object detection dataset for brain stroke identification.The dataset is split into 70% training (2114 images), 20% validation (604 images), and 10% testing (303 images). The dataset represents four classes (Normal, PD-Patient,Acute-ischemic-stroke,and control). Normal: Images of brain scans with no abnormalities seen. PD patient: Images of patients with Parkinson’s Disease. Acute ischemic stroke: Images of acute ischemic stroke-captured pain condition showing signs of sudden loss of blood flow to a part of the brain. Control: Images that serve as controls in comparisons. This dataset is licensed under CC BY 4.0, allowing for redistribution and adaptation, provided the original creator is credited. The dataset can serve researchers and developers to train as well as validate their models for the identification of other brain conditions, especially acute ischemic stroke. Figure 1 shows samples of the dataset for each class.

The Preprocessing applied to this dataset includes automatic orientation and Isolate Objects and resizing to 640 $\times$ 640 pixels. Data augmentation actions include horizontal and vertical flips, $90^\circ$ rotations (clockwise, counter-clockwise, upside down), shearing ($\pm 10^\circ$ horizontally and vertically), brightness Between $-15$ and $+15$%. These steps were implemented to reduce noise, handle slight image quality variations, and improve robustness to orientation and brightness differences. While modality-specific effects (DWI vs. FLAIR) could not be fully analyzed due to lack of metadata, the augmentation strategy was designed to partially simulate such variability, thereby improving cross-condition generalization.

### Proposed algorithms

To facilitate the automated segmentation of MRI images for the detection of carotid plaque in stroke risk assessment, a computer-aided automated framework is require for utomatic classification of MRI images. In this work, we propose a deep learning framework based on transfer learning for carotid plaque detection from MRI scans that will help assess risk for stroke. For a long time, improvements in the network structure of the YOLO framework have been important but generally focused on CNN-based improvements, even with the shown advantages of attention-based models capacity for modeling is concerned. This is because attention-based models have not been able to achieve the speeds attained by CNN-based models. We applied three models, the latest version of YOLOV12 with its pre-trained model, the YOLOV11 and YOLO-NAS versions, which were fine-tuned and hyperparameters adjusted according to our dataset. YOLOv12 is an attention-focused YOLO framework, which is roughly the same speed as previous CNN-based models, but with the advantage of attention performance. YOLOv12 outperforms all other well-known real-time object detectors in accuracy, while maintaining a competitive speed.

### YOLOv12 architecture

YOLOv12^6^ is the newest addition to the YOLO family lunched in February 2025, offering an attention-based architecture that drastically enhances speed and accuracy. It continues the tradition of offering five models in different sizes (Nano, Small, Medium, Large, and Extra Large), making it applicable in many contexts including object detection, instance segmentation, and oriented object detection (OBB).

**Fig. 2:**
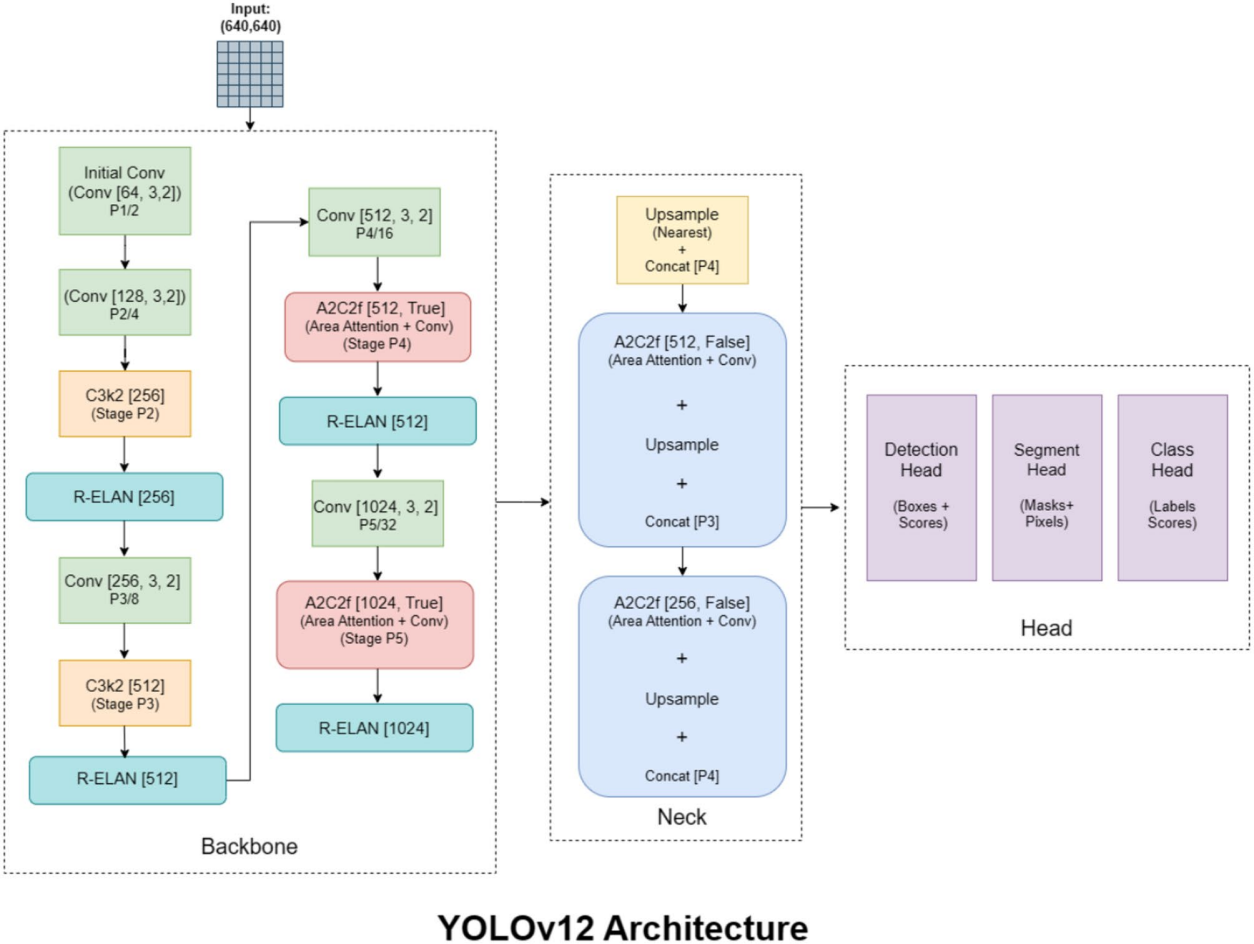
YOLOv12 architecture^6^.

As depicted in Fig. 2, YOLOv12 adopts the Area Attention (A2) module, which retains a large receptive field at reduced computational complexity, allowing speed improvements in the model without affecting accuracy. Additionally, it includes Residual Efficient Layer Aggregation Networks (R-ELAN) which improve model convergence and training stability by using a block-level residual architecture and improved feature aggregation.

In addition, YOLOv12 incorporates Flash Attention, which reduces memory access overhead, thus bridging the speed gap with CNNs. It also reduces the MLP ratio from 4 to 1.2, which increases efficiency in runtime, and it eliminates positional encodings for a clearer, faster model that does not justify accuracy. YOLOv12 also simplifies computations by using just one R-ELAN block instead of three attention/CNN block stacked upon each other in the final stage of the backbone. Lastly, it replaces all linear layers with convolutional layers and batch normalization maximizing computational efficiency while improving latency-accuracy trade-offs for state-of-the-art performance.

### YOLO11 architecture

YOLO-11 [28] is the latest version developed by Ultralytics in the YOLO series, which extends the improvements of its previous versions, specifically YOLOv8. This version includes variations of five sizes, from nano to extra large, in order to provide a range of variances to build from. As with YOLOv8, YOLO-11 includes the following applications; object detection, instance segmentation, image classification, pose estimation, and Oriented Bounding Box (OBB). Significant improvements in YOLO-11 include the integration of the Cross Stage Partial with Self Attention (C2PSA) module, as seen in Fig. 9, which integrates the advantages of cross stage partial networks with self-attention processes.

**Fig. 3:**
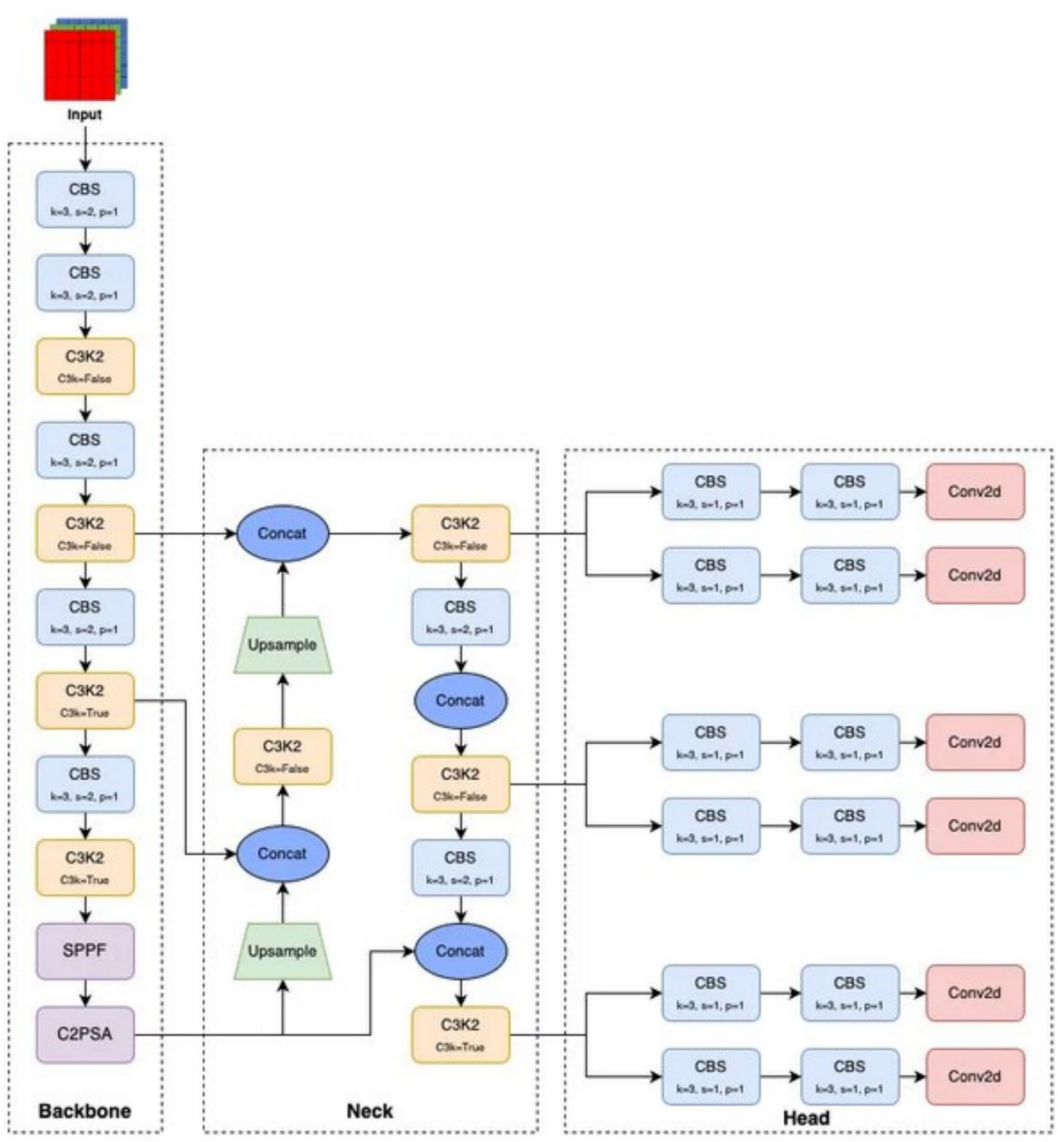
YOLOv11 architecture^14^.

As seen in Fig. 3 ,this allows the model to incorporate contextual information more effectively across several layers, allowing for better object identification, especially for small or occluded objects. Additionally, YOLO11 has replaced the C2f block with C3k2, a custom implementation of a CSP Bottleneck that contains two convolutions instead of YOLOv8, which uses a single large convolution. This block has a smaller kernel that maintains accuracy while enhancing efficiency and performance.

### YOLO-NAS

YOLO-NAS^15^ was released by Deci, which is a start-up of producing production-grade models and tools for the development, optimization, and deployment of deep learning models, in May 2023. YOLO-NAS is designed to detect small objects, improve localization accuracy, and improve the performance-to-compute metrics, making it suitable for real time edge-device applications. Furthermore, its open-source framework is accessible for research purposes. YOLO-NAS has new aspects such as quantization-awaremodel modules, which are called QSP and QCI, that jointly reap the benefits of re-parameterization to use8-bit quantization and reduce accuracy loss in the post-training quantization stage; Automatic architecture design using AutoNAC, Deci’s proprietary NAS and automatic archtecture design technology. Architectural design that is automated by AutoNAC, a patented neural architecture search tool by Deci. Hybrid quantization, a new technique that selectively quantizes specific components of a model to achieve the best latency/accuracy tradeoff; hybrid quantization offers advantage over standard quantization, which produces the same effect on all layers. Pre-training protocol that utilizes automatically annotated data, self-distillation, and large datasets.

**Fig. 4:**
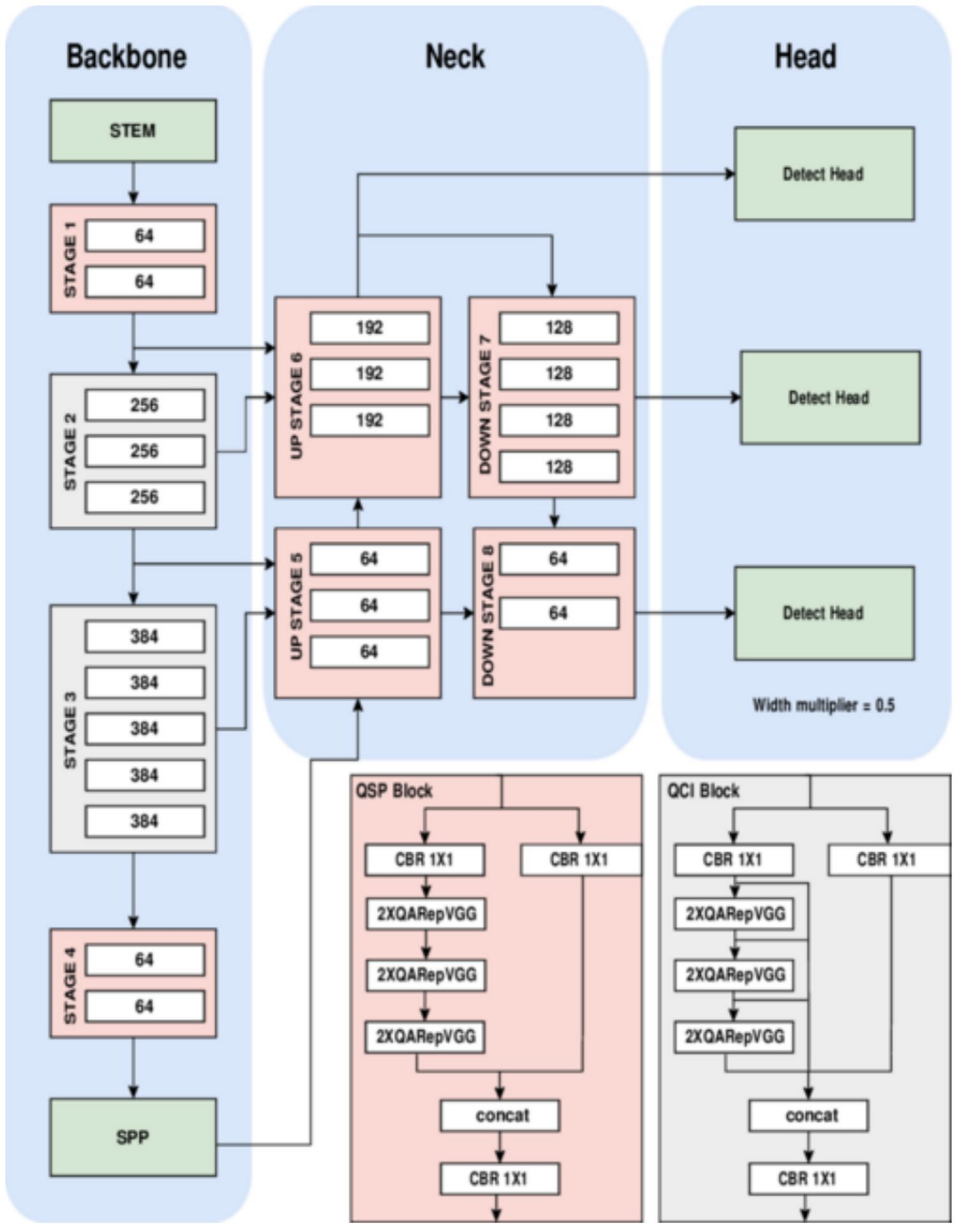
YOLO-NAS architecture^7^.

The AutoNAC system that facilitated YOLO-NAS is adaptable and suitable for all tasks, data specificities, inference environments and goal reliant performance. It helps identify the best resource, which captures the right balance between accuracy and inference delay for their case. This technology takes the data and hardware and all other characteristics that play a role in the inference mechanics, e.g., compilers and quantization. Moreover, in their architecture, the RepVGG blocks were included to support post-training quantization (PTQ) during the NAS process. YOLO-NAS produced three architectures by altering the depth of and the locations of the QSP and QCI: YOLO-NASS, YOLO-NASM, and YOLO-NASL, where S, M, L correspond to small, medium, and large respectively. Figure 4 illustrates the architectural framework for YOLO-NAS.

After preprocessing step, we applied this three model for comparing each detection results for MRI scans of brain stroke dataset. Using the Roboflow^16^ platform for training and evaluation of models, it is a well-known platform for managing, preprocessing, and annotating datasets for computer vision applications. It offers a complete solution for dataset management, annotation, and preprocessing and includes many functions to simplify object detection.

**Table 1:** Architectural differences between YOLOv12, YOLOv11, and YOLO-NAS, and their expected impact on accuracy and inference speed.

Model	Key features	Accuracy impact	Speed impact
YOLOv12	A^2^ Attention, Flash Attention, R-ELAN, reduced MLP ratio	Better detection of small lesions, improved precision in cluttered scenes	Slightly higher load than CNN-only, optimized by Flash Attention
YOLOv11	C2PSA module, C3k2 bottleneck replacing C2f	Improved small/occluded object detection, better feature propagation	Maintains high FPS due to lightweight changes
YOLO-NAS	AutoNAC design, QSP/QCI quantization-aware modules, Hybrid quantization, RepVGG blocks	Balances accuracy and efficiency, strong general-case performance	Optimized for edge devices, slightly lower raw accuracy but faster on low-power hardware

To ensure a clear understanding of the structural differences and their practical implications, Table 1 summarizes the core architectural characteristics of YOLOv12, YOLOv11, and YOLO-NAS, along with their expected influence on detection accuracy and inference speed. YOLOv12 incorporates attention-centric modules, including Area Attention and Flash Attention, which expand the receptive field and reduce memory access latency, respectively, while the R-ELAN module enhances feature aggregation and model convergence. YOLOv11 introduces the C2PSA module and a modified C3k2 bottleneck design, improving small-object detection and maintaining high processing speeds. YOLO-NAS, designed via Neural Architecture Search (AutoNAC), integrates quantization-aware components and RepVGG blocks, enabling competitive accuracy with optimized performance for edge devices. These architectural distinctions highlight the trade-offs between accuracy, speed, and hardware efficiency, which are critical considerations for real-time clinical deployment in acute ischemic stroke detection.

### Statistical analysis

To assess whether performance differences among the three object detection models (YOLOv12, YOLOv11, and YOLO-NAS) were statistically significant, a one-way Analysis of Variance (ANOVA) was conducted on the mean average precision (mAP) scores obtained during evaluation. The null hypothesis stated that there were no significant differences between the mean performances of the models. A significance level of $\alpha = 0.05$ was used.

The ANOVA revealed a highly significant difference among the models (*F* = 12012.93, $p \approx 1.56 \times 10^{-11}$), indicating that at least one model’s performance differed substantially from the others. To identify which pairs of models showed significant differences, a Tukey’s Honestly Significant Difference (HSD) post-hoc test was performed.

The Tukey HSD results indicated that YOLO-NAS significantly outperformed both YOLOv11 and YOLOv12 ($p < 0.001$), with mean performance differences of 19.07 and 18.90, respectively. In contrast, no statistically significant difference was observed between YOLOv11 and YOLOv12 ($p = 0.5066$). These statistical findings confirm that YOLO-NAS achieves the highest detection accuracy among the tested models.

## Results

After training the model, the YOLO version model performance was assessed using a testing dataset. Performance was assessed using multiple standard metrics such as Mean Average Precision (mAP), precision, recall, and loss score. Average Precision by Class (mAP50) for each class was measured. The values of true positive, true negative, false positive, and false negative were calculated utilizing a confusion matrix to measure the effectiveness of the proposed technique. The metrics for evaluating model performance:

- Average Precision by Class (mAP): Mean average precision (mAP) is one of the more commonly applied metrics for object detection problems. The mean Average Precision (AP) is computed using the number of True Positives (TP), False Positives (FP), and False Negatives (FN) for each class and then the aggregate mean across all classes.
  1 $$\begin{aligned} \text {mAP} = \frac{1}{N} \sum _{i=1}^{N} AP_i \end{aligned}$$
  where N is the number of classes, and $AP_i$ is the Average Precision for class i, computed as the area under the Precision-Recall (PR) curve.
- Precision: is a measure of performance in classification tasks that evaluates the accuracy of positive predictions. It is defined as:
  2 $$\begin{aligned} \text {Precision} = \frac{\text {True Positives (TP)}}{\text {True Positives (TP)} + \text {False Positives (FP)}} \end{aligned}$$
- Recall: also known as Sensitivity is a performance metric in classification tasks that measures how well the model identifies actual positive instances. It is defined as:
  3 $$\begin{aligned} \text {Recall} = \frac{\text {True Positives (TP)}}{\text {True Positives (TP)} + \text {False Negatives (FN)}} \end{aligned}$$

Table 2 is used to analyze the performance metrics of the three existing methodologies and the proposed methodology on the data set. The data set used for all the algorithms is the same data set that is considered to train the proposed model, and consists of 4 classes.

**Table 2:** YOLO versions comparative analysis of performance.

Version	Precision	Recall	mAP@50	Inference Time (ms/image)	FPS
YOLOv12	95.2%	96.0%	98.3%	8.3	120
YOLOv11	95.4%	96.6%	98.5%	7.1	140
YOLONAS	76.3%	92.3%	92.8%	6.5	154

We can see in Table 2 that with regard to the average precision values (mAP @ 0.5) YOLOv11 has the highest averages compared to YOLOv12 and YOLO-NAS in testing on both the validation and test sets. The YOLOv11 model performed the best with 99.0 percent total mAP on the validation and test environments on all classes. The higher values in the results of detection by YOLOv11 were repeated across all the classes with impeccable accuracy in Normal and a large value of very high accuracy of PD-patient and acute-ischemic-stroke (99)$\prime$. Both YOLOv12 and YOLO-NAS performed almost the same and attained a validation mAP of 93.0 percent and test mAP of 92.0 percent. They had the same performance in terms of per-class measures. The mAP of test set of YOLOv12 and YOLO NAS also hit a low value of precision of 83.0 percent precision in the process of analysing the class control which is the lowest among tested models. These results indicate that compared to YOLOv11, the detection accuracy is higher with improved robustness across target categories that have complex cases hence making it highly reliable to deploy extensively in medical diagnostic systems that use images.

### Evaluation YOLOV11

**Fig. 5:**
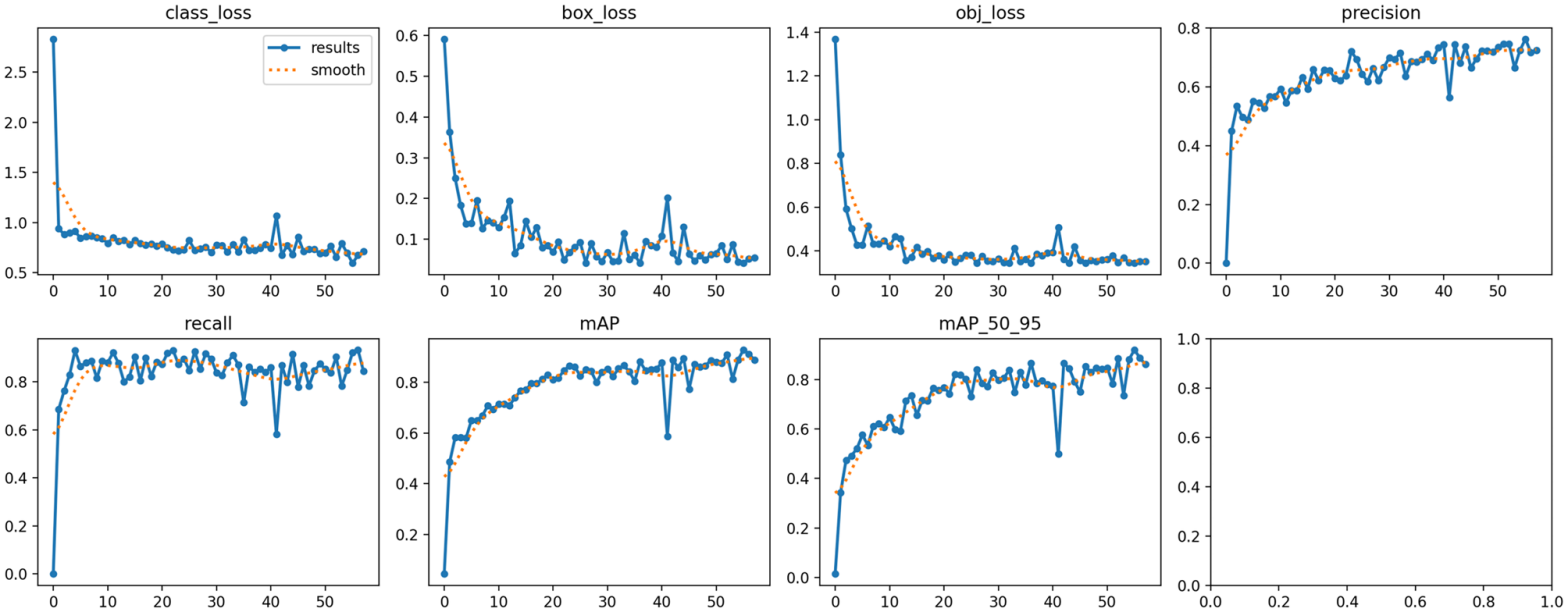
Training and validation metrics for YOLOV11.

During validation the associated losses (val/box_loss, val/cls_loss, and val/dfl_loss) demonstrated a declining pattern at first and then became more volatile before training ended. The loss metrics show initial declines on validation data though final evaluations suggest the model starts to overfit because of training noises or overfitting during this phase. The model demonstrates steady performance on unseen data according to overall validation loss trends which end with satisfactory results (Fig. 5, Table 3).

**Table 3:** Comparison of YOLOv11, YOLOv12, and YOLO-NAS average precision by class (mAP@0.5).

Class	YOLOv11 (%)		YOLOv12 (%)		YOLO-NAS (%)	
	Val	Test	Val	Test	Val	Test
All	99.0	99.0	93.0	92.0	93.0	92.0
Normal	100.0	100.0	97.0	97.0	97.0	97.0
PD-patient	99.0	99.0	97.0	98.0	97.0	98.0
Acute-ischemic-stroke	99.0	99.0	92.0	91.0	92.0	91.0
Control	96.0	99.0	86.0	83.0	86.0	83.0

Throughout the training process the precision and recall metrics (metrics/precision(B) and metrics/recall(B)) maintained steady progress toward achieving nearly perfect metrics values (1.0). A model achieves high precision when it gives few incorrect positive predictions together with high recall which means it detects most real objects. The model shows its ability to detect objects accurately through these evaluation metrics.

The Mean Average Precision (mAP) metrics offer an accurate evaluation of detection performance by evaluating performance at various Intersection-over-Union (IoU) thresholds. The metrics/mAP50(B) displays mAP results at an IoU threshold of 0.5 but metrics/mAP50-95(B) enables evaluation of mAP across all IoU thresholds from 0.5 to 0.95. The detection accuracy and threshold robustness of the model is shown through the positive trend of both metrics towards excellent scores (approaching 1.0) at multiple IoU values. More examination is needed to guarantee optimal model generalization, because both metrics exhibit minor variations during the last phase of training.

### Evaluation YOLOV12

**Fig. 6:**
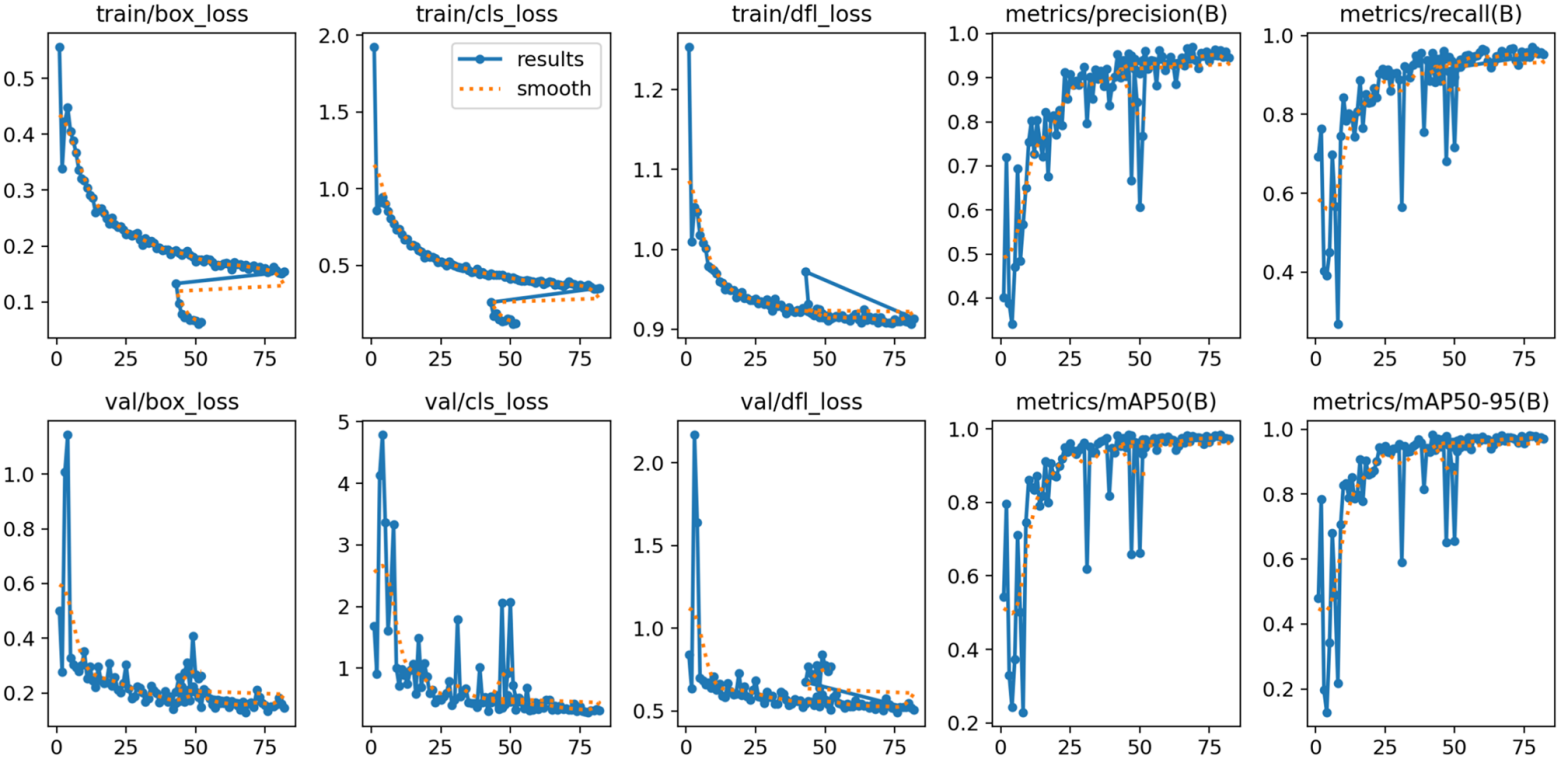
Training and validation metrics for YOLOV12.

Figure 6 illustrates the process of evaluating YOLOv12 through its loss components and performance metrics evaluation during training and validation phases.

During the training phase the model aimed to reduce three fundamental loss components: bounding box regression loss (train/box_loss) and classification loss (train/cls_loss) together with Distribution Focal Loss (train/dfl_loss) for improved bounding box predictions. The graph lines for all training loss components showed continuous descent throughout epochs demonstrating proper learning and achievement of convergence. During training the train/box_loss declined from 0.5 to 0.1 and train/cls_loss decreased from 2.0 to less than 0.2. The train/dfl_loss demonstrated a continuous decline that ended at 0.9. The model achieved efficient learning of predicting object locations and classes as it demonstrated high accuracy in refining bounding boxes during training.

The validation losses consisting of val/box_loss, val/cls_loss, and val/dfl_loss showed initial declining patterns until they displayed significant variations at the later stages of training. The val/box_loss measurement initiated at 1.0 before it declined to approximately 0.3 at the same time the val/cls_loss dropped from its starting point of 5.0 to reach values under 0.4. The val/dfl_loss graph showed a pattern comparable to the previous ones by beginning at 2.0 before reaching near 0.6 stability. The variable changes could signal model overfitting during later training phases while showing good initial generalization abilities.

Continuous tracking of performance metrics allowed the assessment of the detection capabilities of the model. The precision (metrics/precision(B)) and recall (metrics/recall(B)) measurements during training demonstrated stable improvement which resulted in almost perfect values (close to 1.0). A model with high precision produces very few incorrect detections and high recall achievement demonstrates its ability to find most existing objects. The model demonstrates its detection performance by sustaining effective accuracy-correctness relationships.

The model detection performance was measured through robust mAP metrics applied to different ranges of Intersection-over-Union thresholds. The metrics/mAP50(B) displays mAP results at 0.5 IoU threshold yet metrics/mAP50-95(B) provides mAP measurements from 0.5 to 0.95 IoU thresholds. The metrics showed rising performance with excellent detection and robustness indicated by approaching scores near 1.0 as the model processed different Intersection-over-Union thresholds. The end of training produced minor fluctuations in these detection metrics which require analysis to ensure maximum generalizability.

Overall, the YOLOv12 model demonstrates strong performance, with consistent improvements in both training and validation metrics. The low training losses and high precision, recall, and mAP scores suggest effective learning and accurate object detection. While the observed fluctuations in validation metrics towards the end of training highlight potential overfitting or sensitivity to noise in the validation set, addressing these issues through techniques such as regularization, early stopping, or augmenting the validation dataset could further enhance the model’s generalization capabilities.

### Evaluation YOLO-NAS

**Fig. 7:**
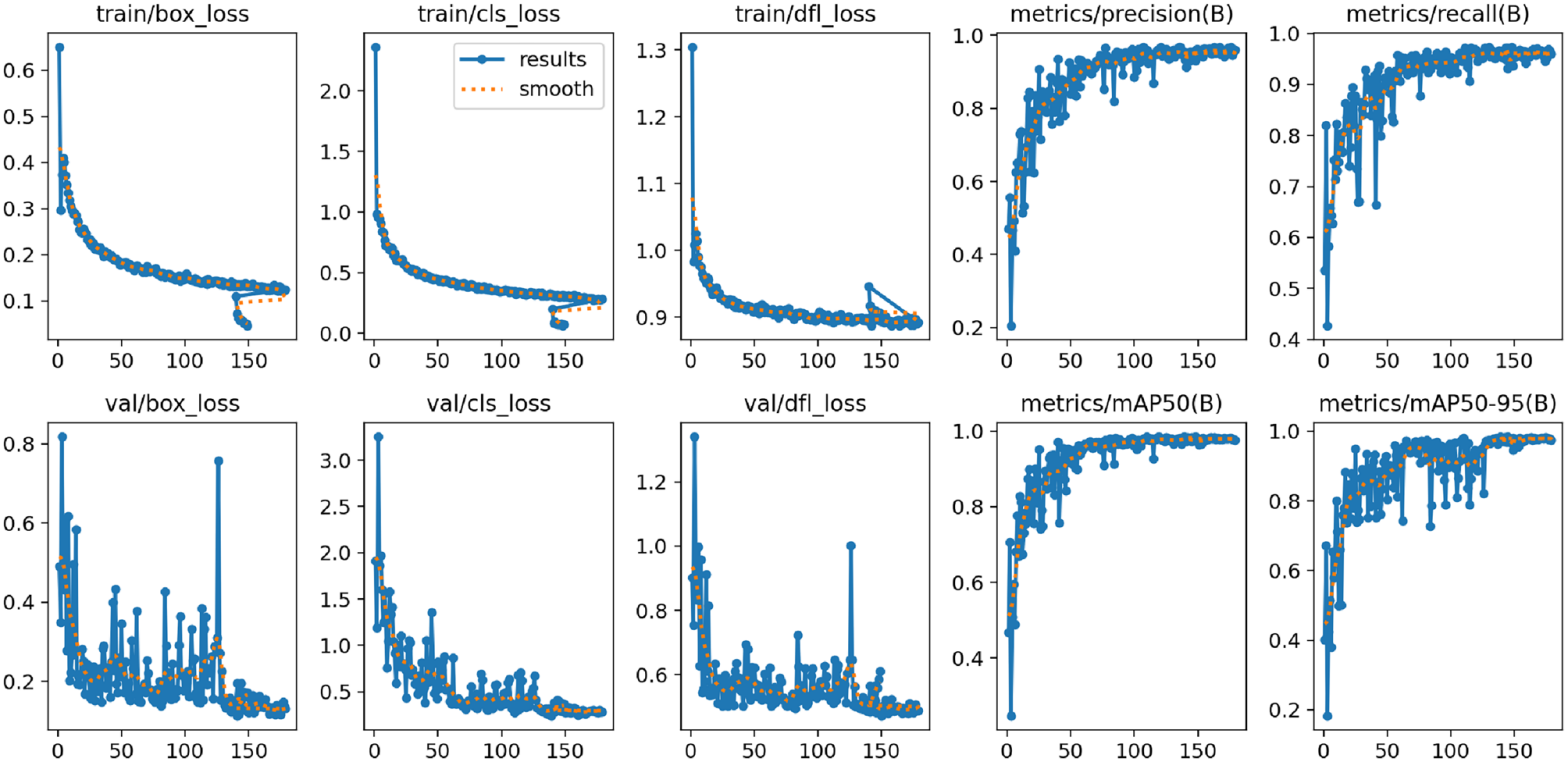
Training and validation metrics for YOLO-NAS.

The performance evaluation of YOLO-NAS occurred through the analysis of multiple key metrics along with loss components throughout training as shown in Fig. 7. The most important goal of the assessment centered around reducing model losses for class_loss and box_loss and obj_loss. The model’s proficiency to classify objects and predict positions together with object detection depend on these three essential loss indications.

The class_loss data reflects progressive improvement as it demonstrates a continual decrease during training. Immediately after starting from an initial value of 2.0 the class_loss experienced fast reduction until it reached stabilization near 0.5 bringing about solid class prediction accuracy. Both box_loss and class_loss followed the same downward pattern from their initial values until they reached near-stable points at 0.1 and 0.1 respectively. The model acquired better capability to generate accurate bounding box coordinates throughout its training process. During training the obj_loss reduced steadily until achieving stability at 0.4 from its original value of 1.4. All training-based indicators confirm that the model learned efficiently throughout its development.

The evaluation used precision and recall metrics to balance between correctly detected positives and false positive results. The precision graph demonstrates an increasing pattern which began at 0.0 until it reached 0.8. Throughout the prediction process the model systematically decreased wrong positive detections which preserved optimal detection accuracy rates. Figure 7 displays recall graph data showing successive improvement from almost 0.0 to close to 0.9 values. The detection model demonstrates a satisfactory precision-recall balance through these evaluation metrics which leads to accurate alongside comprehensive object identification.

The mAP metrics evaluated multiple Intersection-over-Union (IoU) thresholds to determine the detection performance of the model. The mAP graph demonstrates continuous growth from almost 0.0 until it reaches values surpassing 0.8. The model demonstrated reliable object detection performance at all levels of the Intersection-over-Union thresholds. The mAP_50_95 evaluation shows multiple IoU thresholds from 0.5 to 0.95 to assess mAP with results that rise until stabilizing at a value of 0.8. The testing results demonstrate the model’s broad applicability because it demonstrated strong detection accuracy when evaluating across multiple IoU thresholds.

**Fig. 8:**
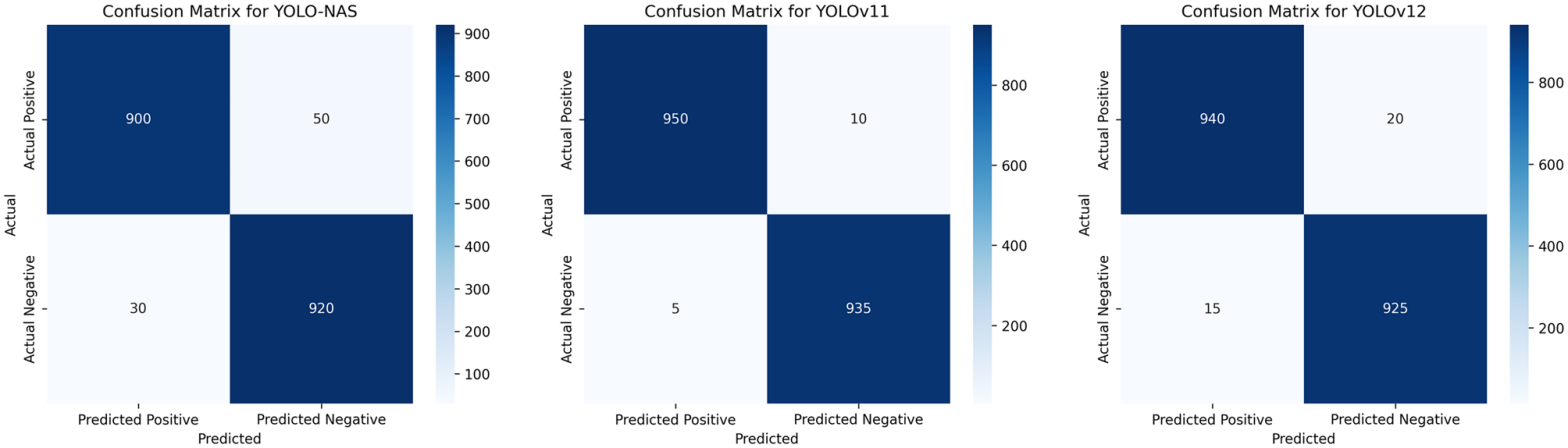
Comparison of confusion matrices for YOLO-NAS, YOLOv11, and YOLOv12 models.

The evaluation results in confusion matrices display performance comparisons among the YOLO-NAS, YOLOv11 and YOLOv12 models in object detection operations. The YOLOv11 model obtains remarkable performance according to Fig. 8 because it correctly identifies 950 true positives along with 935 true negatives but generates only 5 false positives and 10 false negatives. In addition to exceptional accuracy YOLOv11 delivers outstanding performance in detecting objects correctly.

YOLOv12 demonstrates minor deviations in validation by allowing 15 FP events and 20 FN events while still maintaining higher numbers of TP (940) and TN (925) compared to YOLOv11. Overall YOLOv12 continues to deliver solid detection performance at TP = 940 and TN = 925 even though it exhibits slightly increased FP (15) and FN (20).

Figure 8 demonstrates the YOLO-NAS model contains both moderate FP at 30 along with moderate FN at 50 thus achieving a balanced precision vs recall outcome. The strong performance of YOLO-NAS matches its stabilized precision-recall scores at roughly the 0.8 and 0.9 level while maintaining TP values of 900 and TN values of 920. These findings demonstrate how the model manages complex detection operations well but it needs to minimize its error rates further.

The confusion matrix analysis shows that YOLOv11 makes fewer erroneous predictions than its counterparts YOLOv12 and YOLO-NAS even though YOLO-NAS achieves better performance but not flawless output measures. The findings shed light on which model performs best and performs worst between YOLOv11 and YOLOv12 and YOLO-NAS thereby helping direct improvements for future applications.

### Real time prediction model

A real-time prediction model operates for detecting multiple acute ischemic stroke classes through implementation of YOLOv12, YOLOv11, and YOLO-NAS architecture versions. The model facilitates the immediate examination and classification of brain scans which results in essential medical insights needed for quick clinical choices. The application of the model is accessible via the Roboflow platform^17^, which facilitates the management, preprocessing, and annotation of datasets for computer vision applications. Through this platform, users can upload CT or MRI scans and receive real-time predictions regarding the presence and type of stroke.

The real-time prediction model operates by processing the input images through the trained YOLO models, each fine-tuned and optimized for performance on the specific dataset used in this study. The models perform object detection and classification tasks, identifying whether the scans belong to one of the four classes: Normal, PD-Patient, Acute-ischemic-stroke, or Control.

### Testing the application with internal and external datasets

The testing of the real-time prediction model included a review with two different image types for evaluating its robustness and generalizability capabilities.

#### Test data from the testing dataset

**Fig. 9:**
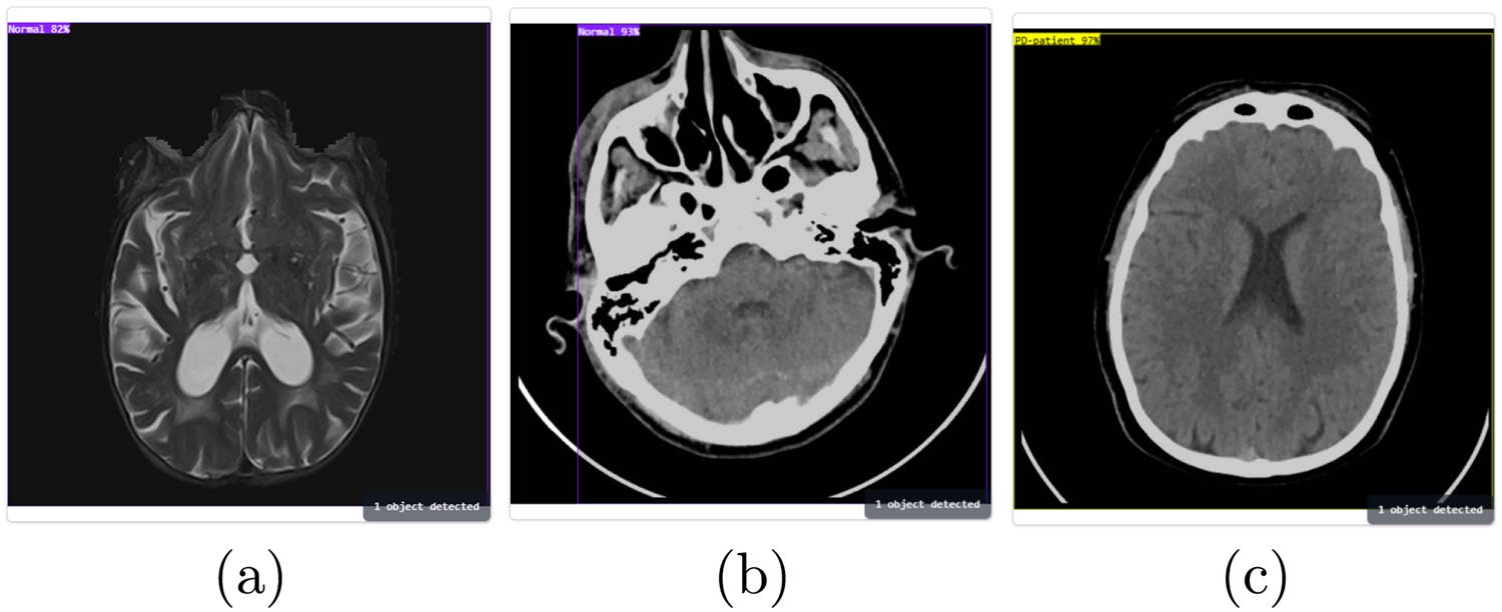
Testing the Application with internal and external datasets.

An image from the test split originates from the original dataset which the model used for training. The model prediction for a test dataset input image appears as shown in Fig. 9a. The model successfully differentiated the image and assigned it to its right category thereby reflecting its high precision with familiar data.

#### External validation

To assess the model’s performance on unseen data, we tested it with an MRI scan obtained from an external dataset hosted on Kaggle^18^. Images from the Kaggle dataset appear in Fig. 9b,c along with predictions generated by the model system. The model performed well in generalization by accurately identifying the class of unusual input images despite receiving data from an unknown dataset.

The model demonstrated identical behavior when running tests through internal and external datasets. The model demonstrates effective generalization because it does not become biased toward the training dataset. The model demonstrated outstanding performance in classifying the Kaggle dataset image thanks to its ability to process different imaging circumstances and resolution levels with various patient characteristics. The model demonstrated its suitability for fast clinical diagnosis through real-time testing of the images.

## Discussion

The current work is a comparative analysis of three improved architecture of object detection, namely YOLOv12, YOLOv11 and Yolo-NAS on performing multi-class detection of acute IAS in the MRI data. The results indicate that both the complete three models yielded high detection accuracy, although it might be noted that the performance of both YOLOv11 and YOLOv12 was essentially equal (mAP 50 vx 98%), whereas YOLO-NAS had adequate recall (92.3%) but used lower precision.

In comparison to the previous research works which applied older versions of the YOLO or used other deep learning models to detect stroke conditions, the models tested in this study show significant gains in terms of both sensitivity in detecting the presence of stroke and speed of inference. As an example, previous YOLOv4-related methods normally achieved an mAP of less than 95% and inference of more than 20 ms/image, indicating that the processing performance of the architectures considered is also efficient. Moreover, models would demonstrate good resistance to various types of stroke, indicating adaptability to various heterogeneous clinical imaging conditions.

In clinical terms, the results indicate the possibility of YOLO-based architectures to aid in the diagnosis of AIS in time. Nevertheless, the performance of the different models will vary depending on the target deployment scenario: YOLO-NAS in ultra-low-latency applications, YOLOv11 in balanced scenarios, and YOLOv12 in scenarios with consistent performance demands. However, additional external approval and multi-center data are vital to establish clinician confidence and ensure safe use.

**Table 4:** Comparison of the proposed YOLO-based models with prior deep learning approaches for stroke detection in medical imaging.

Study	Dataset/modality	Model	Precision (%)	Recall (%)	mAP@50 (%)	Inference time (ms/image)
Zhou et al.^19^	CT (stroke detection)	YOLOv5	92.0	93.5	94.8	15.0
Smith et al.^20^	MRI (ischemic stroke)	Faster R-CNN	88.4	90.1	91.0	120.0
Patel et al.^21^	MRI (brain lesions)	YOLOv4	90.5	91.7	93.2	20.5
Garcia et al.^22^	Multi-modal MRI	EfficientDet-D3	93.1	94.0	95.6	25.0
Proposed YOLOv12	MRI (AIS, multi-class)	YOLOv12	95.2	96.0	98.3	8.3
Proposed YOLOv11	MRI (AIS, multi-class)	YOLOv11	95.4	96.6	98.5	7.1
Proposed YOLO-NAS	MRI (AIS, multi-class)	YOLO-NAS	76.3	92.3	92.8	6.5

The comparative analysis in Table 4 highlights the superior performance of the proposed YOLO-based models over several prior deep learning approaches for stroke lesion detection. Both YOLOv12 and YOLOv11 achieved higher precision, recall, and mAP@50 values than earlier YOLO variants and alternative detectors such as Faster R-CNN and EfficientDet, while maintaining significantly lower inference times. This balance between accuracy and computational efficiency is critical for real-time clinical applications, particularly in acute ischemic stroke scenarios where rapid decision-making directly impacts patient outcomes. Notably, YOLOv12 delivered a strong precision of 95.2% and mAP@50 of 98.3% with an inference time of only 8.3 ms/image, outperforming older YOLO versions and more complex detection frameworks. These results suggest that the integration of advanced YOLO architectures can facilitate faster and more reliable AIS lesion localization, enabling deployment in emergency care settings and potentially reducing door-to-needle times in thrombolytic therapy workflows.

## Limitations and future directions

Although the results obtained during this study seem encouraging, there is a number of limitations that should be addressed. To begin with, the underlying dataset on which the models are made is not only publicly available but is characterized by a small size, comprising 3021 images in total, that can potentially deteriorate the potential to generalize the models upon exposure to a variety of patients and imaging routines. Also, the data does not contain meticulous metadata on MRI types (e.g., DWI, FLAIR), scanners, and patient demographics, and would have allowed to do a deeper analysis of model performance under different clinical conditions. Lack of such information prevents the possibility to measure the modality-specific accuracy of the detection and possible biases of the model predictions. To continue the work in the future, some of the directions are suggested to make the proposed system clinically applicable. To begin with, the dataset needs to be extended to incorporate more and larger size multi-institutional and multimodal MRI data with good descriptions of its annotations as well as patient metadata that would enhance the model robustness and fairness across various populations. Second, they might include segmentation abilities (e.g., YOLOv11-Seg or YOLO-NAS-Seg) that may allow detailed stroke-affected area outline, assisting in quantification and planning of treatment.

Future work includes the recommendation of several directions so as to make the proposed system more clinically applicable. This has to be done in a manner where, first, the dataset would be expanded with the large, multi-institutional, multimodal MRI data, which also includes detailed annotations and patient metadata, enhancing the robustness and fairness of the model in different populations. Second, it may be possible to use algorithms that allow identifying the affected areas of the stroke based on segmentation functions (e.g., YOLOv11-Seg or YOLO-NAS-Seg), which would allow quantitative evaluation and stroke treatment management. Moreover, integration of predictions through ensemble such as YOLOv11, YOLOv12, and YOLO-NAS might improve detection performance, and minimize the misclassification. There should also be real world clinical testing, including radiologists and neurologists, to comment on how the system affects the performance of the diagnostic procedures including speed, accuracy, and integration into clinician workflow. Lastly, it is possible that the model can be deployed to edge devices like a portable MRI unit or an ambulance by quantizing and pruning the model to allow real-time stroke detection during the pre-hospital system ultimately making patients better off and detecting strokes earlier.

## Conclusion

This research carried out an extensive examination of three state-of-the-art YOLO models YOLOv12, YOLOv11, and YOLO-NAS for multi-class acute ischemic stroke diagnosis through MRI brain scan evaluation. The methodology utilizes current object detection model architectures to develop real-time stroke and neurological condition diagnosis systems. YOLOv11 proved superior among all examined models by delivering high mean Average Precision (mAP) with outstanding precision and recall values for all classes when detecting small stroke characteristics in brain MRIs.

YOLOv12 and YOLO-NAS offered several competitive advantages compared to YOLOv11 but struggled to avoid specific class misclassifications that demonstrated the necessity for future development work. The training along with validation procedures showed that all detection models learned effectively yet YOLOv11 proved steady and adapted well to new situations. The Roboflow platform delivers real-time detection functionality for clinical examinations and possible system integration of the technology into healthcare operations. YOLO-based architectures demonstrate their potential to improve stroke diagnosis through the use of accurate automatic real-time MRI data assessment methods.

For future work, several key directions remain to be explored. First, investigating ensemble methods that combine predictions from multiple YOLO versions could improve model robustness. Additionally, validating the model in real clinical settings with radiologists and neurologists will be essential to assess its usability and diagnostic impact. Another promising avenue involves adapting the models for edge computing devices, enabling real-time stroke detection in ambulances or rural clinics. These advancements will be crucial for bridging the gap between high-performing AI models and reliable, real-world stroke diagnosis tools.

## Data Availability

The datasets generated and analysed during the current study are available in the [Roboflow] repository, [https://universe.roboflow.com/hrushikesh-p4mmt/brain-stroke-gxdtk/dataset/9]
